# Colorophone 2.0: A Wearable Color Sonification Device Generating Live Stereo-Soundscapes—Design, Implementation, and Usability Audit

**DOI:** 10.3390/s21217351

**Published:** 2021-11-05

**Authors:** Dominik Osiński, Marta Łukowska, Dag Roar Hjelme, Michał Wierzchoń

**Affiliations:** 1 Department of Electronic Systems, Norwegian University of Science and Technology, NO-7491 Trondheim, Norway; dag.hjelme@ntnu.no; 2 Consciousness Lab, Institute of Psychology, Jagiellonian University, 30-060 Kraków, Poland; marta.lukowska@doctoral.uj.edu.pl (M.Ł.); michal.wierzchon@uj.edu.pl (M.W.)

**Keywords:** Colorophone, sensory substitution, color sonification, multimodal perception, wearable device, assistive device, human–computer interaction

## Abstract

The successful development of a system realizing color sonification would enable auditory representation of the visual environment. The primary beneficiary of such a system would be people that cannot directly access visual information—the visually impaired community. Despite the plethora of sensory substitution devices, developing systems that provide intuitive color sonification remains a challenge. This paper presents design considerations, development, and the usability audit of a sensory substitution device that converts spatial color information into soundscapes. The implemented wearable system uses a dedicated color space and continuously generates natural, spatialized sounds based on the information acquired from a camera. We developed two head-mounted prototype devices and two graphical user interface (GUI) versions. The first GUI is dedicated to researchers, and the second has been designed to be easily accessible for visually impaired persons. Finally, we ran fundamental usability tests to evaluate the new spatial color sonification algorithm and to compare the two prototypes. Furthermore, we propose recommendations for the development of the next iteration of the system.

## 1. Introduction

Visual-to-auditory sensory substitution devices (SSDs) aim to compensate for sensory function loss by delivering information acquired by the visual channel (i.e., via camera or distance sensors) through hearing [1]. Surprisingly, the first electronic SSD of such type was first developed in 1897 by Noiszewski [2]. Nonetheless, there is still no SSD that has been widely accepted broadly by the blind community [3,4,5]. It contrasts with the recent research results that indicate significant potential for SSDs for non-invasive rehabilitation of the visually impaired [4,6,7] stemming from brain plasticity. According to the multimodal/supramodal brain organization hypothesis [8,9], the human brain operates as a flexible, task-oriented system. Namely, it has been repetitively demonstrated that its organization is more function- than modality-specific. For example, in the case of visual loss, brain areas normally dedicated to visual input processing receive sensory input from other modalities that serve the same function (i.e., spatial cognition) [10]. Importantly, the neuroplastic changes are not restricted to a critical period of brain development and can also occur in adults. This suggests that with proper training, interpretation of the translated sensory information may become intuitive and effortless over time, and a new quality of perceptual experience might be developed [11]. Together with observed progress in the electronic systems field [12], these encouraging results fuel the intensive growth in the development of novel SSDs in recent years [13,14]. However, the color-to-sound coding devices seem relatively unexplored. To the authors’ knowledge, only 11 systems realize color sonification [15,16,17,18,19,20,21,22,23,24,25], while only six provide real-time sonification of the acquired color information [18,19,22,23,24,25] (see Section 4—Existing Color-to-Sound SSDs for details). One of these was the first version of the Colorophone system [25].

### Colorophone

**Figure 1:**
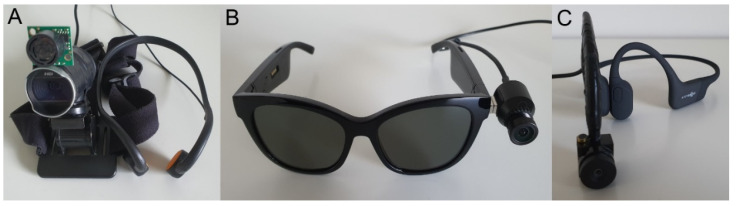
Subsequent versions of the Colorophone system: (**A**) Colorophone 1.0 with a camera mounted on the headband and headphones; (**B**) Colorophone 2.0 with Bose Bluetooth audio sunglasses; (**C**) Colorophone 2.0 with Aftershokz bone-conducting headphones.

The main goal of the Colorophone project [26] is to develop an affordable, wearable SSD that will enhance perceptual and cognitive capabilities of the visually impaired. We aim to achieve this by providing auditory information about color and distance in an intuitive form. We have performed a preliminary evaluation of the previous version of the Colorophone system conducted on blindfolded, sighted participants. It showed promising results in color and object recognition as well as spatial navigation tasks [25]. Nonetheless, the first prototype had multiple issues regarding its usability and functionality. Firstly, in the previous implementation, we used a standard webcam attached to a headband, which appeared to be bulky. The numerous cables connecting the camera and headphones to the processing unit decreased its usability by reducing users’ comfort. Moreover, the generated sounds were artificial sine signals and low-pass filtered white noise that were unpleasant to hear over a prolonged time. Finally, the main functional issue was the fact that the system delivered a nonspatial sensory output by processing the information only from a single area of interest—the focal point of the head-mounted camera. Therefore, we decided to address the above-mentioned disadvantages by developing the next version of the system. Crucially, the new version provides spatial information about colors by sonifying the whole horizontal line of camera pixels. Additionally, we developed a dedicated opponent color space that mimics the human visual system’s opponent process, providing more intuitive color categorization, and aims to enhance the auditory color recognition of yellowish colors. Moreover, we improved the appearance and aesthetics of the second version of the system (see Figure 1). A supplementary video example presenting the operation of both versions of the system and a spectrogram of the generated signals can be found in https://youtu.be/fWeKpGMFlmk (accessed on 30 October 2021).

## 2. Design Considerations of SSDs Development

Here, we briefly discuss several design considerations regarding the development of color-to-sound SSDs. We will individually address issues enumerated by Kristjánsson et al. [1]. Additionally, we will comment on several features that can be used to benchmark electronic travel aids (ETAs) presented by Dakopoulos and Bourbakis [27] that can also be helpful while developing new SSDs.

The first design principle states that only critical information about the environment should be conveyed to avoid the risk of sensory overload. Therefore, the designed conversion method should avoid filling up the whole sensory channel; instead, only the chosen parts of the accessible information space should be used.

Another inherent challenge in creating visual-to-auditory SSDs is the mismatch in the information bandwidth between visual and auditory sensory channels. It is estimated that we perceive two or three orders of magnitude more information through vision than through audition [1]. This disproportion is also reflected by the comparison of the number of neural fibers in the optic and auditory nerves. The optic nerve has over one million fibers [28], while the auditory one has over 30,000 fibers [29]. However, the bandwidth of perceptual experience does not reflect the amount of information that can be accessed consciously [30], which is promising information for SSD developers. The human limitation in the amount of consciously attended information can potentially reduce the influence of mismatch in bandwidth. Nonetheless, there still is a firm conviction that SSDs should be task-focused [1]. In our case, the main task for the designed SSD will be color recognition based on auditory color representation [25].

The next design requirement of no interference with other perceptual functions is in our opinion the most difficult to address while designing visual-to-auditory SSDs. It is impossible not to interfere with the acquisition of surrounding sounds while conveying the auditory information via an SSD. However, this interference can be limited by using open-ear headphones and designing the color sonification method to limit the masking effects.

The following requirements are related to design toward usability. The device should be conveniently wearable and easy to operate without using the hands. However, when adjustment in settings is required, the users should be able to straightforwardly change the intensity of the auditory signals according to their demand to perceive environmental or substituted information.

The last design requirement involves a spatiotemporal continuity of coded information. Perception is a continuous process that does not involve a snapshot of the environment [1]. Therefore, the designed method for color sonification should allow for continuous transitions of the auditory information based on spatial and temporal color changes.

## 3. Why Color?

Although it is challenging to provide a clear definition of what color is [31], it is easier to identify why this attribute of visual perception has an evolutionary function. Color vision provides organisms with important sensory information about the environment that increases their chances of survival [32], by supporting object identification and enhancing the ability for object-ground segmentation [33,34]. Interestingly, the utility of color information for object recognition is greater for medium and low resolutions than for higher resolutions [35]. Still, color is an elusive concept, which cannot be easily described to someone who has never experienced it [31]. A blind person can haptically access information about the shape or distance to the object. However, there is no natural way to access color information via other sensory modalities. This results in the exclusion of visually impaired people from this feature of the perceptual experience, leaving language as the only widely used medium of conveying information about colors.

## 4. Existing Color-to-Sound SSDs

There are many SSDs that convert visual information to auditory signals [36,37,38,39,40,41,42,43] (see [44] for a detailed review). However, as mentioned before, there are relatively few systems that realize color sonification. Here, we provide short descriptions of color sonification methods implemented in existing systems. Detailed descriptions of the color sonification methods and comparison of experimental results are presented in [25,33]. Information about systems’ features, including camera integration, real-time processing and spatialized sound, are shown in Table 1.

ColEnViSon [15] categorizes information about color to one of 10 color categories and associates them with sounds in the following way: red as electric jazz guitar, yellow as a synth drum, brown as a guitar fret noise, orange as a bird tweet, green as a shamisen, blue as a vibraphone, violet as a glockenspiel, black as guitar harmonics, gray as a celesta, and white as a music box. The lighter intensities of the same color are represented as notes on higher scales of the same instruments.

Hue Music [16] categorizes color information as one of the eight distinct hues. Color categorization is implemented using an RGB color model and rounding up to the value of 255 for every value above 127 and rounding down to 0 for every value equal to or above 127. Every hue value is associated with a timbre. Hue values used for timbral associations are red, yellow, green, cyan, blue, magenta, and white. The white color component is represented by silence.

Musical Vision [17] is an image sonification system that uses an RGB color model. Color saturation is coded as volume, and pitch changes represent the spatial location of the pixels. The system reduces color information by discarding the lowest intensity colors and converting RGB values of every pixel into three instruments or chords.

SoundView [18] uses the HSV color model. Grayscale colors are represented by low-pass filtered white noise. The filter’s cut-off frequency proportionally depends on brightness levels. Twelve color components are represented by band-pass filtered white noise. The bandwidth of the filters is inversely dependent on color saturation; thus, saturated colors are perceived as tones.

Creole [19] uses CIELUV color space. Color component values are nullified if the amplitude of the color component does not exceed 0.2 of the maximum color value in the processed image. Color intensity is represented by sound loudness. The Creole system represents red as the male vocal vowel sound of “u”, yellow as a C major chord (1047, 1319 and 1568 Hz), green as the male vocal vowel sound of “i”, blue as a C minor chord (262, 311 and 392 Hz), black as a low-pitched tone of 110 Hz, and white as a high-pitched tone of 3520 Hz. Desaturated colors are represented by band-passed white noise (100 to 3200 Hz).

EyeMusic [20] operates similarly to the vOICe system [36], where the acquired image is processed column by column from left to right, constructing a soundscape. The luminance is coded as loudness, and the vertical position of the processed pixel is associated with pitch changes. However, the sounds used in the EyeMusic system are recordings of musical instruments, and every instrument represents a different color. Red is represented by a reggae organ, yellow by string instruments, green by Rapman’s reed, blue as brass instruments, and white as a choir. Various timbres represent the color information, and the pentatonic scale is used for pitch-elevation coding. Only the dominating color for every pixel is played.

Cavaco et al. [21] used the HSV color model. Loudness represents the value, and hue is mapped to pitch (i.e., when the light wave frequency decreases from violet to red, the sound frequency increases). The color saturation is represented by timbre changes from a sinusoid for the lowest saturation to a square wave for the highest saturation.

KromoPhone [23] provides three different color sonification modes, where the default and most advanced is the RGBYW mode. Color intensity is mapped onto the sound volume. Subsequent colors are represented as follows: red as a high-pitch trumpet tone in the right ear, yellow as a high-pitch ukulele tone in the left ear, green as a medium-pitch violin tone in the right ear, and blue as a low-pitch trumpet tone in the left ear. White is represented by high pitch, gray by middle pitch, and black as low pitch—all centrally heard sounds.

Eyeborg [22] codes color saturation as sound volume and continuously transposes light frequencies into sound frequencies. Red is coded by a sound frequency of ≈364 Hz that increases with the light frequency changes up to ≈608 Hz for violet.

The first version of Colorophone [25] uses a dedicated RGBW color space. Color component intensity is coded as loudness. Red is represented by a high-pitch tone of 1600 Hz, green as a middle-pitch tone of 550 Hz, blue as a low-pitch tone of 150 Hz, and white as white noise. The white color component is calculated as a minimum value of RGB color components, which is afterward subtracted from all input RGB values. Black is represented by silence. Amplitudes of color components are perceptually linearized.

The See ColOr system [24] represents colors by using an HSL color model and associates hues in the following way: red is represented by an oboe, orange by a viola, yellow by a pizzicato, green by a flute, cyan by a trumpet, blue by a piano, and purple by a saxophone. The transition between sounds is calculated as a linear relationship between consequent hue values. The pitch of a selected instrument depends on the saturation value. Additionally, darker colors are coded with double bass, while singing voices code brighter colors.

To sum up, there are only four SSDs that provide camera integration and real-time color sonification (two crucial features while considering the use of the system as a blind aid). Only See ColOr [24] uses sound spatialization; however, it does not provide continuous sound output, breaking the spatiotemporal continuity of generated signals. In the next section, we will analyze the possibilities of developing a color sonification method that will enable the generation of intuitive, continuous, and spatialized auditory representation of visual information.

## 5. Color Sonification

Since the goal of color sonification is to convert information from visual to auditory channels that are inherently different, the necessary preliminary step is to specify the conversion system’s purpose. In SSDs used for visual rehabilitation of the blind, the primary function of color sonification algorithms is to provide intuitive information about color by a sound that will enable auditory color perception and recognition. This section builds on the initial design considerations regarding auditory color space development presented in [45].

### 5.1. Design Considerations Regarding Auditory Color Space

The existing color sonification methods applied in SSDs can be divided into three categories: the first category contains systems that directly associate light frequency with sound frequency [21,22], the second category systems use associations between a predefined color category and the presented sound [20,46] (e.g., the sound of a choir represents the white color). In other words, every color (from a limited palette—usually only a few colors are covered) is represented by an associated sound, which imposes strict color categorization, and sharp transitions between sounds corresponding to different colors. The third category of systems uses basic color components associated with sound components [19,23,25,33]. In such systems, auditory color representation is constructed from many sound components merged into a single auditory stimulus. The devices from the last category provide satisfactory results in experiments related to complex color recognition [25]. Additionally, the approach allows the utilization of the multidimensionality of color experience in the auditory color representation. Therefore, we decided to use the last approach while designing the new color sonification method.

#### 5.1.1. Psychophysics

Since sight and hearing show different psychophysical characteristics, we implemented an inverted Stevens’s power law [47] for the auditory channel. It compensates for the nonlinear volume perception of the human auditory system. The information about the color intensity is preprocessed by the inverted Stevens’s power law function, which then is annulated by the influence of the human auditory system.

#### 5.1.2. Cross-Modal Correspondences

Cross-modal correspondences are natural associations between different sensory modalities, such as bright objects being loud [48]. The usage of cross-modal correspondences enhances the performance of auditory color recognition [19]. Although finding a universal mapping of various sensory modalities remains ambiguous, we can utilize existing research results as a guideline in designing the color sonification method. The first intuitive mapping between a color component and a sound component would be mapping the intensity of the color stimuli to the intensity of the sound stimuli. More intensive colors will be associated with higher volume sounds. We chose to associate color components with corresponding sound frequencies on the basis of the pitch–chroma relationship described in [19].

#### 5.1.3. Number of Color Components

When selecting the number of color components to be represented by a sonification algorithm, we should bear in mind that if this number is too large, it will be difficult for a naïve user to remember and recognize all the color–sounds associations. However, if the number is too low, a user will not have the necessary variety in the auditory signal to be able to recognize a color change. Our preliminary tests indicated that the RGBW color space [25] allowed satisfactory auditory color recognition of 14 tested colors (black, white, red, pale red, green, pale green, blue, pale blue, yellow, pale yellow, violet, pale violet, cyan, and pale cyan), but the recognition of colors near yellow (orange, olive green) appeared challenging. Although these two colors are visually perceived as saliently different, their sound representations were perceived as very similar. In addition to improving discrimination between yellowish colors, adding the yellow color component into the developed color space has other advantages. Firstly, red, yellow, green, and blue are assumed to be elementary colors called unique hues, and the subjective appearance of any other color can be composed of these unique hues [49]. Moreover, the yellow component is central in opponent process theory [50]; thus, the yellow–blue axis is present in many advanced color spaces. Therefore, we consider the yellow color component to be necessary for our color sonification design. Black remains a unique color component because the information about this color, which effectively means lack of any light, can be analogously conveyed by silence—the lack of any sound. The proposed color space of five color components plus black strongly reminds of color component definition from the Natural Color System (NCS) [51] that is entirely based on the phenomenology of human perception.

### 5.2. Color Spaces

NCS is one of the various color spaces that define the conventions of coding information about color with numerical values. CIELAB and CIELUV are often used, where uniform color spaces are based on the opponent process theory [31]. However, neither of the mentioned color spaces have focal colors as color axes. CIELAB does not have focal red, blue, or green anywhere close to the corresponding color axes, and CIELUV has the most significant deviation from the axes for green, yellow, and red color components [50]. By focal colors, Kuehni [52] defines the ideal representatives of a given basic color name. While designing auditory color space based on previous considerations, we need to use a color space based on opponent process theory, where color axes are as close as possible to focal red, yellow, green, and blue. We propose to call the color space equipped with the features described above as RYGBW, where letters represent the following color components: red, yellow, green, blue, and white.

#### 5.2.1. RYGBW Auditory Color Space

**Figure 2:**
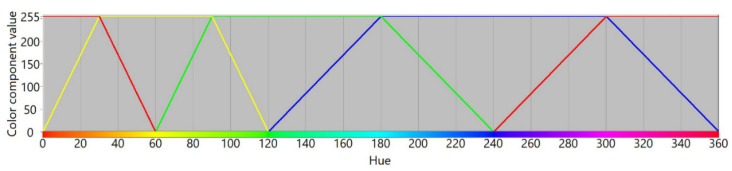
Transitions between chromatic color components in RYGBW color space.

The RYGBW color space is constructed based on the RGB color model by calculating the W component as a minimum value of all RGB components that is subtracted from the original RGB values. Thereafter, the Y component is dependent on the amplitudes of R and G components in a way that makes the transition of color components between red and yellow, and yellow and green similar to the transitions for other unique hues. To visualize color component variability, we plot respective RYGBW values for color transitions presented using an HSL color model. The transitions between fully saturated colors for the whole hue spectrum are presented in Figure 2. According to the design idea, the yellow component becomes a new dimension in the constructed color space, similar to red, green, and blue.

**Figure 3:**
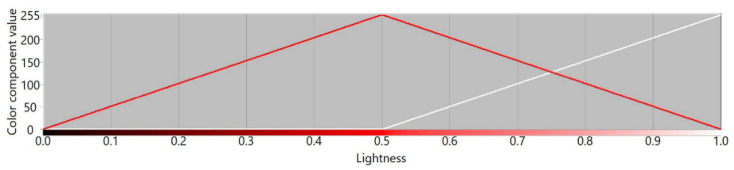
Example of the color transition for a single-color component from black through red to white in RYGBW color space.

**Figure 4:**
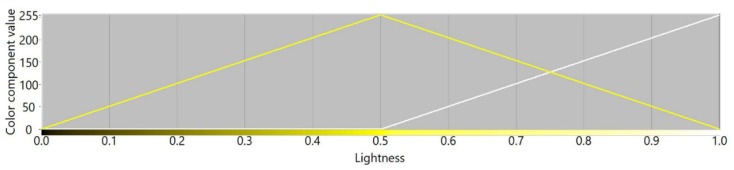
Example of the color transition for a single-color component from black through yellow to white in RYGBW color space.

**Figure 5:**
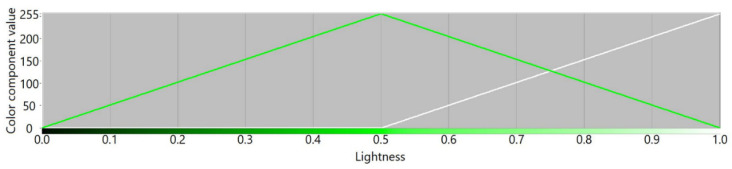
Example of the color transition for a single-color component from black through green to white in RYGBW color space.

**Figure 6:**
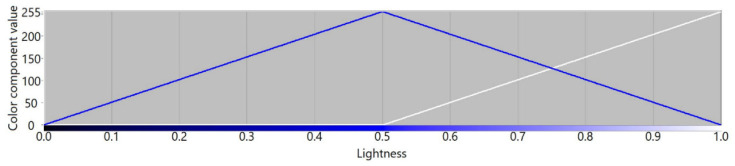
Example of the color transition for a single-color component from black through blue to white in RYGBW color space.

**Figure 7:**
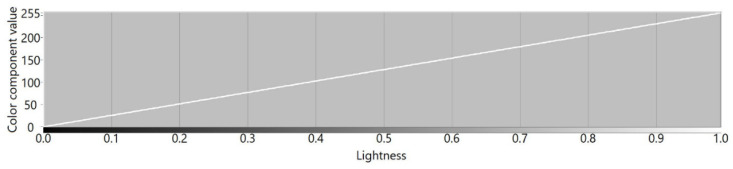
Example of the color transition for a single-color component from black to white in RYGBW color space.

Figure 3, Figure 4, Figure 5 and Figure 6 present transition profiles for individual color components from black, through fully saturated color, to white. When the white color component increases, the value of other color components decrease respectively. Figure 7 presents color transition for non-saturated colors from black through gray to white.

#### 5.2.2. Sounds Associated with Color Components

While choosing sounds corresponding to colors, we used the following guidelines:

● The sounds should:
  ○ Be pleasant for the user [25];
  ○ Be calibrated in amplitude corresponding to the maximal color intensity to provide equal loudness for every sound component;
  ○ Have higher difference in frequency than the critical bands to avoid masking effects [53];
  ○ >Be preferably perceptually equally spaced in pitch [54]; and,
  ○ Be associated with colors on the basis of chosen cross-modal correspondences (i.e., blue—low pitch, green—middle-low pitch, yellow—middle-high pitch, red—high pitch).
● White should be coded by a sound with no characteristic primary frequency such as white noise or rainfall.

Since we know which sound pairs will be presented together, we can choose to simultaneously present only dissonant pairs of sounds, which positively influences the recognition of sound components [55]. Importantly, to preserve the sound continuity, we considered only musical instruments that allow for seamless looping of the used sound samples such as the violin or trumpet. The ceiling frequency was 1027 Hz to maintain a high resolution in sound localization possibilities [56,57] (for details, see Section 5.3.1 Sound Localization) and avoid high-pitch sounds that are perceived as unpleasant [58]. The lowest used frequency was empirically chosen to avoid excessive vibration of bone-conducting headphones. Based on these considerations, we decided to choose the associations between the color and sound components presented in Table 2.

### 5.3. Spatial Color Sonification Algorithm

The human ability of sound source localization opens a possibility for the development of a spatialized color sonification algorithm. This may be realized by applying the sonification method described above to code the color information from a larger number of areas of interest (zones) located on the processed image into auditory signals. However, it requires the parallel computation of color to sound conversions for multiple zones using high-quality digital waveforms and applying functions necessary for sound spatialization.

#### 5.3.1. Sound Localization

When a sound reaches our ears, we use subtle differences in sound timing, intensity, and spectral composition to determine sound source location [59]. The angles describing the sound source location in the polar coordinates system are called azimuth for the horizontal plane and elevation for the vertical plane. The difference in distance between our ears and the sound source results in interaural time difference (ITD), and the shadowing effect produced by the head causes interaural intensity difference (IID). The ITD and IID together are called binaural localization cues [59]. The ITD is the dominant binaural cue—it is a major cue for the localization of low-frequency sounds, but it also contributes to high-frequency sound localization [60]. It is assumed that the diffraction effect of an average human head is negligible for sound waves of frequencies below 1 kHz and that IID is too small to facilitate sound localization for frequencies below 1500 Hz [59]. The minimum audible angle (MAA) parameter is used to investigate the human sound localization ability. The MAA defines the smallest perceptible difference in the position of a sound. In the sighted population, it has been demonstrated that for wideband stimuli and low-frequency tones presented in the frontal position in the horizontal plane, the MAA is on the order of 1° to 2° [59,61]. However, the MAA depends on the position of the sound source in the horizontal plane and a sound frequency. Importantly, the ability to localize a sound source decreases rapidly for frequencies between 1050 and 2500 Hz [56].

Moreover, sound localization is more precise in the frontal (i.e., when one is located frontally to the sound sources) as compared to the lateral (i.e., when one is located laterally to the sound sources) position. Namely, it has been demonstrated that an average error in absolute localization for a broadband sound source is about 5° for the frontal and about 20° for the lateral position [59].

For moving sound sources, the minimum audible movement angle (MAMA) is used to determine the limits of sound localization abilities. It has been demonstrated that changes in sound frequency similarly influence both MAA and MAMA [62]. Namely, the MAMA is smaller for signals below 1050 Hz than for higher frequencies [57]. However, the relationship is nonlinear (i.e., in the range between 250 and 1050 Hz, it takes a U-shape and increases for frequencies above 1050 Hz; for details, see [56]). Another experiment that investigated MAMA for a broadband noise source moving at the velocity of 20°/s showed MAMA values on the order of 2° for azimuth angles in the range of 0–40° and 4° for the angle of 80° [63].

The highest base sound frequency used in the Colorophone system is 1027 Hz. It has been chosen to meet the limit of accurate sound localization based on ITD [56] and dynamic spatial resolution [57]. Here, we propose the spatialized sound implementation based only on ITD. For calculating ITD, we use a frequency-independent model of a wave propagating around a sphere expressed by Woodworth’s formula [64]:

(1) $$ITD=\frac{a}{c}\left(sin\theta +\theta \right),$$

where a is the radius of the sphere, c is the speed of sound (343 m/s), and θ is the lateral angle. For the average head radius value, we used 87 mm from an estimation of a spherical head model based on anthropometry [64]. It is essential in the context of SSD development that sound localization ability is preserved while using bone-conductive headphones [65].

#### 5.3.2. Zone Size Determination

Based on the camera’s viewing angle and image resolution, sound generation parameters, and limits of spatial auditory resolution, we can calculate the minimal zone size and consequently the number of sonification zones. Since the number of zones influences the calculational load of the system, it is important to configure the zones’ sizes and positions in a way that sounds coming from adjacent zones should be potentially distinguishable. For low-frequency tones arriving from the frontal position, the MAA corresponds to an ITD differences of 10–20 µs. The chosen sound sampling frequency will determine the minimum temporal resolution to time difference corresponding to one sample. The standard sampling frequency recommended by the Audio Engineering Society for professional digital audio is 44,100 Hz [66]. The time difference between samples for 44,100 Hz frequency is ≈23 µs, which matches the ITD difference corresponding to MAA. For the horizontal camera resolution of 640 pixels and 90° field of view (FoV), the zone size corresponding to a 1° viewing angle is 7.1 pixels. However, taking into account MAMA, which will probably be more suitable considering enactive head movements, the minimum zone size can correspond to 2–3° of the camera’s viewing angle (i.e., ≈14–21 px for the camera’s FoV of 90°). Nonetheless, it is essential to remember that MAMA increases for larger azimuth values; therefore, while using a wider FoV, the minimal zone size will increase to 4–6°, which corresponds to ≈28–43 pixels. To sum up, we chose the minimal zone size of 14 px for the central azimuth values and gradually increased the zones’ sizes for the higher azimuth values corresponding to lateral zones.

## 6. The Colorophone 2.0 SSD

**Figure 8:**
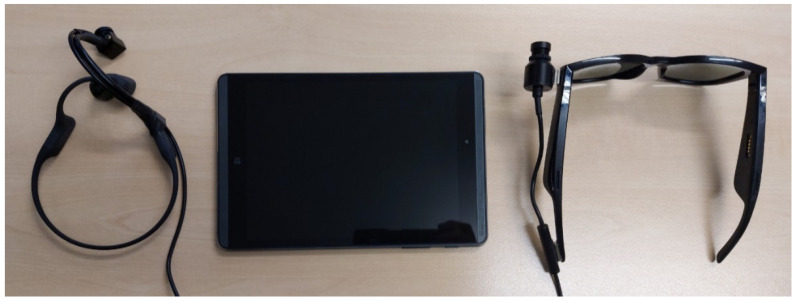
Two realizations of the Colorophone 2.0 system together with the processing unit in the form of a Windows tablet.

Here, we propose two implementations of the Colorophone system that consist of a Bluetooth camera and headphones, and a processing unit in the form of a Windows tablet (see Figure 8). We have also implemented two software interfaces—one designed for researchers and the second one for visually impaired users. Both versions use the same sonification algorithm that performs visual data acquisition, data processing, and sound generation.

### 6.1. Wearable Prototypes

We have built two wearable versions of the system: one based on Bose Frames audio sunglasses [67] and the second based on Aftershokz Aeropex [68] bone-conducting headphones. The comparison of the relevant headphone parameters is presented in Table 3.

**Figure 9:**
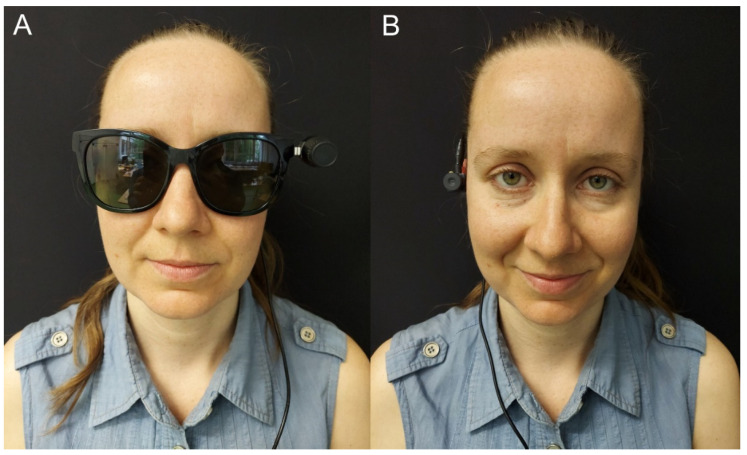
Two realizations of a wearable camera-headphone prototype: (**A**) Bose Frames; (**B**) Aftershokz Aeropex.

Both cameras are equipped with an OmniVision OV2735 image sensor and a USB-C 2.0 interface. The field of view of both cameras is 90°. The camera used in the prototype based on the Bose Frames uses a two-point magnetic connection (on the left side), while the camera used in Aftershokz Aeropex is mounted on the right side of the headphones with a flexible arm and tape (see Figure 9). The applied mounting solutions enable free interaction with functional buttons of both prototypes.

### 6.2. Processing Unit

The processing unit is an HP 608 Pro tablet equipped with an Intel Atom x5-Z8500 with Intel HD Graphics (1.44 GHz, up to 2.24 GHz using Intel Burst Technology, 2 MB cache, and 4 cores), 4 GB LPDDR3-1600 SDRAM, 64 GB embedded Multi Media Card (eMMC), 7.86-inch diagonal capacitive multi-touch, FHD QXGA BrightView WLED UWVA (2048 × 1536), and a 21 Wh lithium–polymer battery. The tablet’s external dimensions are 137 mm by 207 mm by 8.35 mm, and it weighs 420 g. The installed operating system is Windows 10.

### 6.3. Software

**Figure 10:**
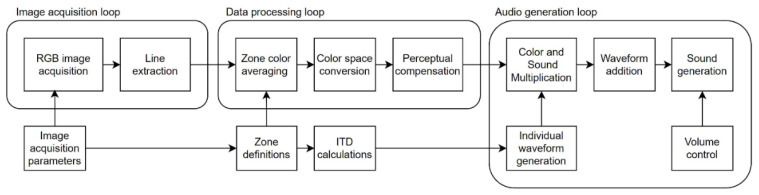
Functional block diagram of the sonification algorithm implemented in the Colorophone 2.0 system.

The software has been developed using LabVIEW 2020 by National Instruments [69] with an add-on Vision Development Module. LabVIEW is a programming environment that allows for relatively easy system development and integration of various peripheral devices. The developed system acquires images via a USB camera, codes visual data into waveforms, and outputs sound via Bluetooth headphones. The functional block diagram showing the operations of the system is presented in Figure 10.

The system operates in three parallel loops responsible for image acquisition, data processing, and audio generation. Although dependent on the data transfer between them, these loops communicate in a way that ensures stable operation of the whole system. For example, if the image acquisition loop automatically reduces the frame rate in response to poor lighting conditions, it does not interrupt the data processing loop’s function and, consequently, the audio generation loop. During the start of the system, all loops are initialized with configuration parameters that influence various loop functions.

#### 6.3.1. Image Acquisition

The first loop in our processing pipeline is responsible for the continuous acquisition of RGB images from an external USB camera. Before starting the loop operations, a dedicated function identifies possible video operation modes of a connected camera. Then, a chosen video mode of 640 × 480 pixels is used to initialize the connection with the camera. Then, images are acquired continuously at the rate of 30 frames per second. Then, the image is converted to an array of pixel values, and a horizontal line is extracted from the array and sent via a local variable to the next data processing loop.

#### 6.3.2. Data Processing

The second data processing loop reads the configuration data calculated based on image acquisition parameters and zone definitions. These data are used for setting zone boundaries, determining which pixels should be included for every zone. Then, the averaging of color information for every zone is performed. The output RGB information is converted into RYGBW color space, and a compensation ensuring perceptual linearity in the auditory channel is applied. The current implementation data processing loop calculates color values in parallel for 15 zones and sends the information about RYGBW parameters for every zone to the following audio generation loop.

#### 6.3.3. Audio Generation

The last loop generates auditory signals based on ITD calculations and processes the information about color data received from the previous loop. During program initialization, .wav files containing sound samples corresponding to every color component are loaded into the memory. Then, 80 individual waveforms are generated based on data received from the ITD calculation function. These waveforms are looped in order to preserve sound continuity. Then, the amplitudes of waveform values are multiplied by their respective color component amplitudes for every zone and by the volume control value. The single audio generation loop iteration takes 30 ms; thus, the maximum information processing time of the whole system is 60 ms. Every sound loop iteration creates a new soundscape that is sent to the default Windows audio output device.

#### 6.3.4. Interface for Researchers

**Figure 11:**
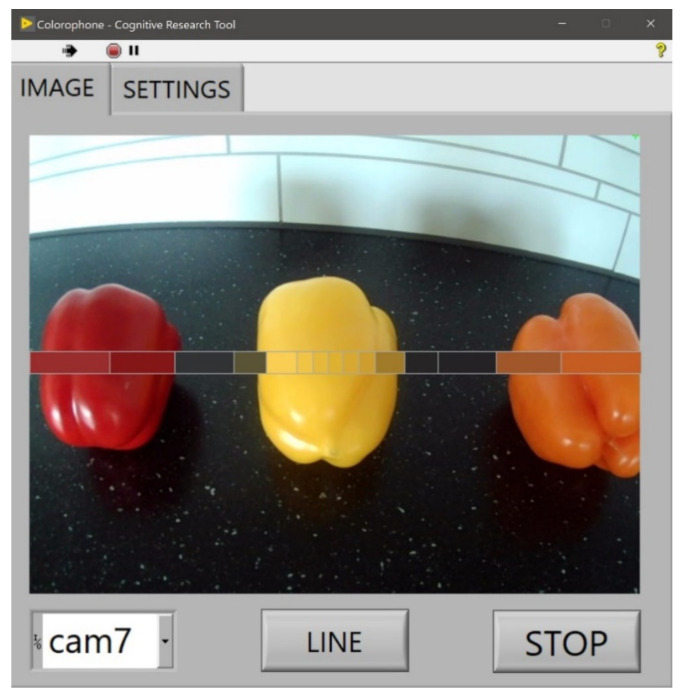
The Colorophone interface for researchers—the main image tab of the GUI.

Figure 11 presents the dedicated graphical user interface (GUI) for researchers. The acquired image is presented together with a visualization of the averaged color information for every processed zone. The GUI allows access to multiple configurative functions, such as choosing the camera and switching between color sonification modes for nonspatial and spatial processing.

**Figure 12:**
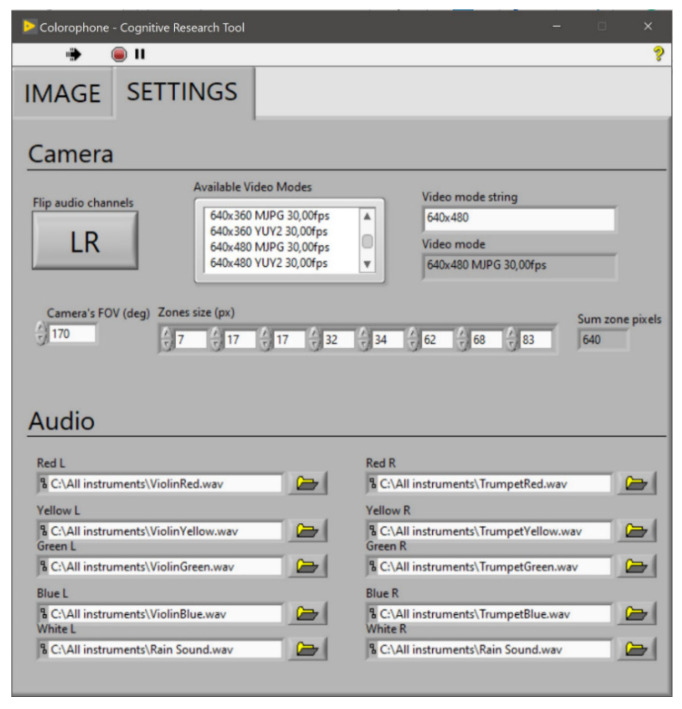
The Colorophone interface for researchers—settings tab of the GUI.

The settings tab (Figure 12) contains more configuration options. Here, one can flip left and right channels. This option can be used for non-standard camera mounting that involves rotation of the camera. It is also possible to choose various video parameters of camera operation by identifying the desired image resolution from the list of available video modes and using a video mode string to extract the chosen option. Another variable enables the user to set the camera’s FoV and define sizes of individual zones. The control sum of the pixels from all zones is displayed to prevent errors in zone sizes definition. The program also allows for the choice of sound samples used individually for every channel by the sonification algorithm.

#### 6.3.5. Interface for Blind Users

**Figure 13:**
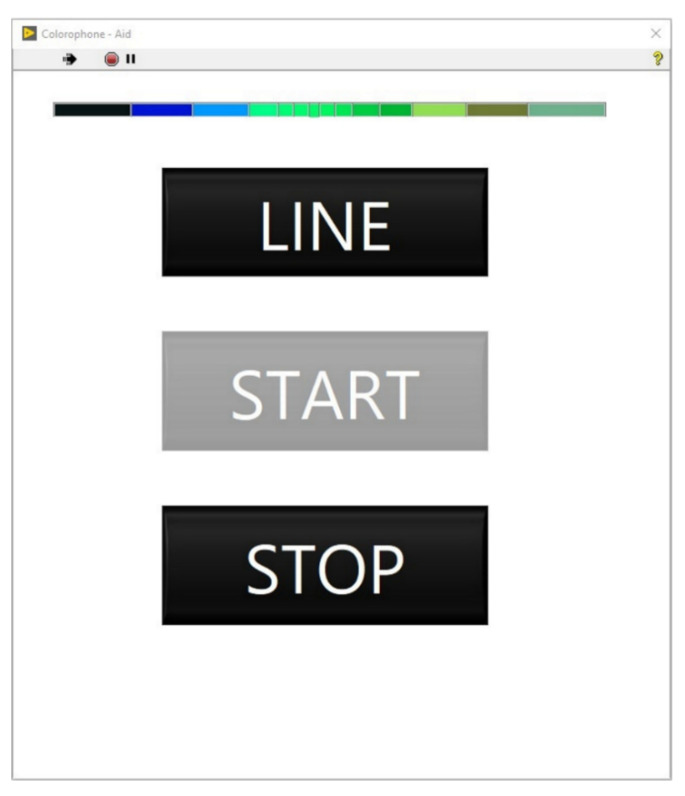
The Colorophone high-contrast GUI.

The second developed interface is implemented both in the form of a graphical as well as an auditory user interface. The GUI has been designed to reflect the need for a high-contrast display and contains a limited number of buttons (Figure 13). *START* and *STOP* buttons are used for turning on and off the sonification process—the *LINE* button switches between the point and zone processing mode. The mode change is also reflected in the appearance of the color box above the *LINE* button (i.e., it switches between presenting the color information for nonspatial and spatial modes). The auditory user interface operates by using interaction cues—a user explores the whole screen haptically, and when they touch a button localization on the screen, a voice command reads the button name. The second touch of the button activates the chosen option, and a voice command of “*Going to …*” is played for the user together with the button’s label. For example, when a user slides their finger over the *LINE* button, the first played message is “*Line*”; after a click, the *LINE* mode is activated, and the message “*Going to line*” is played. The system also generates speech-based error messages. When the camera gets disconnected during the operation of the system, the user receives the following message: “*Houston, we have a problem. Check the camera cable and restart the app*”.

### 6.4. Applied System Settings

For the purpose of the system evaluation, it has been set up in the following way: violin sounds were associated with the colors on the left side of the image, while trumpet sounds coded colors on the right side of the image. Using two different instruments provides an extra cue regarding sound spatialization. The color of the central zone was associated with simultaneously played violin and trumpet sounds. The sound of rainfall represented white. To investigate the possibility of using the whole angular range of sound localization, the camera FoV was set to 170°, so the camera’s image was “stretched” in the auditory domain to cover almost the whole azimuth sound variability. We defined 15 zone sizes ranging from 14 to 83 pixels (i.e., one central and seven on the left and right side).

## 7. Evaluation of the System

**Figure 14:**
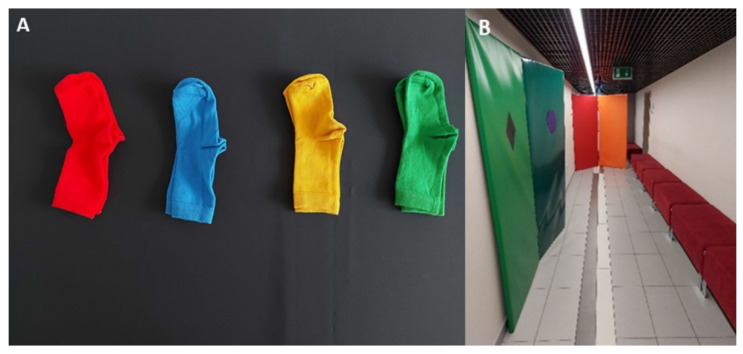
Setup used in the two tasks of the usability audit: (**A**) peripersonal space task (color recognition, object localization, pointing, and grasping); (**B**) extrapersonal space task (colorful object localization, route planning, locomotion, object’s shape recognition).

A usability audit was conducted to initially evaluate the functionality of the Colorophone 2.0 and prepare for in-depth, multidimensional usability tests with visually impaired users. The audit was conducted by an interdisciplinary research team consisting of a UX specialist, a cognitive science researcher, and a qualitative studies researcher. All three specialists were sighted and gave written consent to participate in the audit. Importantly, the UX specialist has never had contact with the device before, whereas the two other experts were experienced in sensory substitution research. We aimed to compare the two prototypes, assess the intuitiveness of the color-to-sound mapping, analyze differences in the two sonification area modes (i.e., nonspatial and spatial), track sensorimotor contingencies (i.e., the regularities in how sensory stimulation depends on the activity of the perceiver/user [70]) required by the device, and evaluate the system usability to solve everyday life tasks (i.e., natural object recognition, color identification, reaching, and locomotion). The audit was conducted in three steps, starting with a free exploration phase when the specialists were allowed to interact with the system without a defined goal. Then, a task testing the Colorophone usability in the peripersonal (i.e., within hand reach) space was administered. In the task, a user was positioned in front of a table, where a few pairs of colorful objects of the same size were located (see Figure 14A). The user was asked to find an object of a given color, point on its location, and grasp it. All three specialists underwent the task under four conditions resulting from the combination of the two variables: prototype (Bose vs. Aftershokz) and sonification area mode (nonspatial vs. spatial). Then, the second task, testing the system usability to solve tasks in extrapersonal (i.e., behind hand reach) space was administered (see Figure 14B). The task was to find a green wall in a corridor and then recognize the shape of an object located on the wall. Given that the specialists did not undergo any formal training of the Colorophone’s usage before the audit, we can conclude that they managed to fulfill all tasks to a satisfactory level. Namely, after a while, they were able to recognize and find the object of a given color in peripersonal space and navigate slowly in the indoor environment. Given users’ expertise level and sample size (i.e., the three specialists), we employed a qualitative approach to identify potential problems and propose an adequate improvement. Here, we present the main conclusions from the audit with recommendations for further studies.

Firstly, after the exploration and the first task, we concluded that the Bose-based prototype is more suitable because the Aftershokz implementation is unstable (i.e., it was moving during exploratory head movements, constantly changing the position of the sampling camera attached to the headphones). In effect, users cannot develop efficient sensorimotor contingencies because of the variability of the sampling device in relation to their body and external space. Namely, they were unable to correctly interpret where the signal was coming from and, as a result, could not locate objects in space based on the sonified color information. However, we found that the Bose headphones are restricting access to external sounds more than the Aftershokz bone-conducting ones. Therefore, we suggest to either develop the Bose-based prototype or to use the Aftershokz but with the camera attached separately to avoid instability.

Secondly, we found that the applied transformation of the camera field of view into a sound space was confusing. Namely, the camera’s FoV is 90°, which is converted to 170° in the sound space. This transformation creates serious confusion as to where an object is located in physical space. Additionally, in both prototypes, the camera is located on the head side, which causes even more confusion concerning objects localization in reference to the sampling device and body. Both of the above-mentioned problems have implications for the ability to locate sound sources in space solely on the basis of information provided by the Colorophone. Moreover, it poses a risk of a long-lasting egocentric reference frame (ERF) recalibration as in case of a hemispatial neglect; patients suffering from this medical condition omit objects located in their left visual field due to an altered central body axis sensation. In our case, the ERF recalibration may result in a sensation as if the central body axis is shifted (i.e., translated or rotated) toward the camera location [71]. More specifically, a user learns the relationship between the object’s location in space and the location of the sound in the auditory space provided by the Colorophone (i.e., acquires new sensorimotor contingencies). Later, when we remove the device, the egocentric reference frame is recalibrated, and the user may have a problem with a correct localization of stimuli based on auditory information that is no longer mediated by the device. This is because they continue to apply the correction acquired over the training with the device. This is potentially dangerous, as it can have a long-lasting effect as a prismatic adaptation (i.e., egocentric reference frame recalibration treatment applied in the hemispatial neglect patients) [72,73]. Importantly, in case of visually impaired users, a readaptation to an adequate egocentric reference frame might be difficult due to restricted access to visual information [74]. Apart from confusion and the risk of egocentric reference frame recalibration, the current spatial transformation and the lateral camera location might cause headaches and nausea, as it was reported already after two hours of using the device by all three users. However, the reported aversiveness of the white color sound representation might also contribute to the observed headache. Nonetheless, we suggest to change the visual-to-auditory spatial transformation algorithm to provide proper correspondence between an objects’ location and its auditory space (i.e., the auditory space should be restricted exactly to the camera FoV). Additionally, we recommend locating the sampling device (i.e., camera) on a central body axis (e.g., in the middle of the forehead or between eyes). Optionally, a hand-held version of the camera could be enabled to avoid neck muscle tension after long-term head-mounted Colorophone prototype usage.

Thirdly, the comparison of the nonspatial (i.e., one sonification zone) and spatial (i.e., multiple sonification zones) modes revealed that they might serve different functions. Namely, the former could be more efficiently used in peripersonal (i.e., close) space tasks (e.g., object location within reach and grasping), whereas the latter could be more efficiently used in extrapersonal (i.e., far) space tasks (e.g., object location behind reach, route planning, and navigation). Moreover, the modes seem to complement each other to support some functions; for example, in the indoor navigation, the spatial mode could be used to have a glimpse of the whole surroundings and locate an object that one wants to move toward, and then, the nonspatial mode might serve as a ‘rope’ enabling to keep direction during locomotion toward the chosen direction. However, the current implementation of the spatial mode should be reconsidered to account better for the auditory spatial resolution of visually impaired users. This is because in the audit, the users found it difficult to identify which sound is heard in which spatial zone. It might be potentially even harder for visually impaired users, since some of them could demonstrate lower auditory spatial resolution (i.e., minimum audible angle) [75,76,77,78]. Therefore, further studies concerning the optimal number and size of the zones in the spatial mode should be conducted. Additionally, individual differences in MAA should be considered to customize the multiple zones mode, and further investigations should identify which functions are best supported by which mode, as well as how the modes can complement each other to support everyday life activities of visually impaired users.

Finally, we identified several issues concerning color-to-sound mapping. The users found the sound representation of white color distracting and potentially aversive (i.e., motivating to avoid exploration of bright environments). Given the omnipresence of white color in natural and indoor environments, we should consider choosing a less attention-consuming and more pleasant sound corresponding to white color or reduce the device sensitivity to this component. Moreover, black representation as silence was also found as problematic, especially when trying to recognize shapes of black objects. So, adding a sonified black color representation should be considered or introducing a function that would reverse white and black color auditory representations. Additionally, we recognized a problem with variability introduced by changing lighting (i.e., introduced by user’s shade, light source changes, or by observation angle changes). In the visual modality, top–down processes provide color constancy [79], but the current implementation of the Colorophone sonification algorithm does not account sufficiently for the lighting-induced variability. Importantly, it significantly impacts the ability to identify objects based on the sonified color information, since the way a given object sounds is radically affected by the lighting context. The lack of a color stability problem has to be solved to support the Colorophone-mediated color cognition.

In summary, the current implementation of the Colorophone system seems promising as a tool to support both color and spatial cognition. However, the above-mentioned issues concerning visual-to-auditory spatial transformation, the sampling device location, white and black color sound representation, and color stability have to be solved to increase Colorophone usability. Importantly, we should bear in mind that the audit was conducted without systematic training, and the experts cannot be perceived as SSD super-users. It is possible that a prolonged practice with the Colorophone will solve some of the above-mentioned problems by decreasing cognitive load, embodying the device, and enabling an intuitive usage strategy [80]. Therefore, studies with well-trained visually impaired users in their natural environment might shed more light on Colorophone usability.

## 8. Conclusions and Further Work

We have successfully developed and initially evaluated a wearable, real-time, spatialized color sonification system. The system uses a dedicated opponent color space that mimics some of the low-level functions of the human visual system. The device generates continuous, rich, and relatively pleasant (i.e., as compared to other SSDs) soundscapes. In contrast to the previous version of the system, the current version provides spatial color sonification for 15 zones based on a pixel line covering the whole horizontal FoV of the camera. Sine signals and white noise has been replaced with more pleasant sounds of instruments and rainfall. We have proposed two new realizations of the device that use mini cameras, providing a relatively discreet and modern look. The system allowed users to fulfill the tasks of finding an object of a given color in peripersonal space and navigating slowly in the indoor environment without conducting prior systematic training.

The disadvantages of the system’s current implementation include issues connected to the lateral mounting of the camera and scaling of the camera’s FoV, the camera’s inability to accurately compensate for various light conditions that results in object’s colors inconstancies and potential identification problem. Importantly, the system’s implementation does not provide information about the distance to the objects. However, this issue has been addressed by developing an additional Colorophone visual echolocation function introduced in [81].

Further work will include addressing the problems pointed out by usability audit and further tests of the next version of the system with visually impaired users. We will focus primarily on improving the spatial sonification algorithm. Firstly, we will consider results concerning the auditory spatial resolution of visually impaired users [75,76,77,78] to define the number of zones and their sizes in the spatial mode. Secondly, we will implement assumptions of the auditory scene analysis [82,83] to define how many auditory streams can be experienced as separate at the same time with the spatialized version of the Colorophone. Finally, we will evaluate several configurations of the multiple sonification zone mode with the visually impaired users to determine the multiple sonification zones mode. Additionally, we aim to develop a more informative black sound representation and less aversive white sound representation to increase the signal-to-noise ratio and improve colorful object detection on a bright or dark background, as well as to account for an illumination difference between daytime and nighttime. Moreover, we plan to investigate the whole Interactive System (composed of User, Environment, and Technology, according to the Personalized Inclusive SSDs Design model that is currently under development). So, not only will we focus on the device development, but also, we will consider how to adapt the user’s environment and account for individual differences between users to enhance Colorophone usability for everyday life tasks.

## Figures and Tables

**Table 1:** Comparison of color-to-sound sensory substitution systems.

System/Author	Camera Integration	Real Time	Spatial Sound
ColEnViSon	No	No	No
HueMusic	No	No	Yes
Musical Vision	No	No	Yes
SoundView	No	Yes	No
Creole	No	Yes	No
EyeMusic	Yes	No	Yes
Sofia Cavaco et al.	Yes	No	Yes
Eyeborg	Yes	Yes	No
KromoPhone	Yes	Yes	No
Colorophone 1.0	Yes	Yes	No
See ColOr	Yes	Yes	Yes

**Table 2:** Chosen associations between color and sound components.

Color Component	Sound Frequency (Hz)	Note	Sound Type
Red	1027	C6	Musical instruments
Yellow	647	E5	
Green	408	G#4	
Blue	256	C4	
White	-	-	Rainfall

**Table 3:** Comparison of the two open-ear headphones used for prototyping.

Headphones	Bose	Aftershokz
Weight (g)	50	26
Battery life (h)	Up to 5.5	8
Charging time 0–100% (min)	60	90
Waterproof (IP)	No (IPX2)	Yes (IP67)
Retail price ($)	249.95	159.95

## Data Availability

Data sharing not applicable.
